# Multi-Band Scattering Characteristics of Miniature Masson Pine Canopy Based on Microwave Anechoic Chamber Measurement

**DOI:** 10.3390/s25010046

**Published:** 2024-12-25

**Authors:** Kai Du, Yuan Li, Huaguo Huang, Xufeng Mao, Xiulai Xiao, Zhiqu Liu

**Affiliations:** 1 Qinghai Provincial Key Laboratory of Physical Geography and Environmental Process, College of Geographical Science, Qinghai Normal University, Xining 810008, China; 20211028@qhnu.edu.cn (K.D.); maoxufeng@yeah.com (X.M.); 2 State Forestry and Grassland Administration Key Laboratory of Forest Resources and Environmental Management, Beijing Forestry University, Beijing 100083, China; lsdjl@bjfu.edu.cn; 3 Key Laboratory of Tibetan Plateau Land Surface Processes and Ecological Conservation (Ministry of Education), Qinghai Normal University, Xining 810008, China; 4 Southern Qilian Mountain Forest Ecosystem Observation and Research Station of Qinghai Province, Huzhu 810500, China; 5 Laboratory of Target Microwave Properties, Deqing Academy of Satellite Applications, Deqing 313200, China; xxl@dasa.net.cn (X.X.); lzq@dasa.net.cn (Z.L.)

**Keywords:** forest canopy, scattering contribution, radar profiles, incidence angle, polarization decomposition

## Abstract

Using microwave remote sensing to invert forest parameters requires clear canopy scattering characteristics, which can be intuitively investigated through scattering measurements. However, there are very few ground-based measurements on forest branches, needles, and canopies. In this study, a quantitative analysis of the canopy branches, needles, and ground contribution of Masson pine scenes in C-, X-, and Ku-bands was conducted based on a microwave anechoic chamber measurement platform. Four canopy scenes with different densities by defoliation in the vertical direction were constructed, and the backscattering data for each scene were collected in the C-, X-, and Ku-bands across eight incidence angles and eight azimuth angles, respectively. The results show that in the vertical observation direction, the backscattering energy of the C- and X-bands was predominantly contributed by the ground, whereas the Ku-band signal exhibited higher sensitivity to the canopy structure. The backscattering energy of the scene was influenced by the incident angle, particularly in the cross-polarization, where backscattering energy increased with larger incident angles. The scene’s backscattering energy was influenced by the scattering and extinction of canopy branches and needles, as well as by ground scattering, resulting in a complex relationship with canopy density. In addition, applying orientation correction to the polarization scattering matrix can mitigate the impact of the incident angle and reduce the decomposition energy errors in the Freeman–Durden model. In order to ensure the reliability of forest parameter inversion based on SAR data, a greater emphasis should be placed on physical models that account for signal scattering and the extinction process, rather than relying on empirical models.

## 1. Introduction

Forest ecosystems, as major terrestrial carbon reservoirs, play a crucial role in mitigating global warming through carbon absorption and storage [1]. Masson pine (*Pinus massoniana*) is a key afforestation species in southern China, characterized by its extensive distribution and contribution to forest carbon sequestration [2]. However, optical remote sensing faces challenges in effectively monitoring the growth and health of Masson pine forests owing to the frequent cloud cover and rainfall in southern China. Microwave remote sensing, with its long wavelength, cloud-penetrating capability, and sensitivity to moisture content, presents advantages and potential for estimating biomass and monitoring health status in Masson pine forests [3,4].

The estimation of Masson pine parameters using microwave remote sensing requires a clear understanding of target scattering characteristics. Indoor and outdoor microwave observation experiments are crucial for investigating vegetation microwave scattering properties and enhancing mechanistic understanding [5]. Outdoor observations accurately reflect the vegetation’s scattering characteristics in natural environments but are susceptible to unpredictable noise from weather conditions and the surrounding environment, complicating detailed quantitative analysis. In contrast, indoor observation experiments, conducted in a controlled environment, allow for precise measurement and quantitative analysis of microwave scattering from vegetation, thereby enhancing the interpretation of scattering characteristics. Comparing the pros and cons, the indoor observation shows greater advantages in understanding scattering behaviors, although miniature scenes are required due to platform size limitations.

Currently, scholars have conducted microwave chamber experiments to measure forest scattering characteristics across different observation bands. For the X-band, studies have pointed out that leaves contributed the majority of backscattering and extinction and further proposed that components in the tree structure with sizes similar to wavelengths could contribute more backscattering energy [6,7,8]. For the C-band, Zoughi et al. suggested that the depolarization effect of branches and petioles was more important than that of leaves, and the size, shape, water content, and relative position of branches had a significant effect on the contribution of backscattered energy [9]. Paris described the crown scattering properties in peach trees through backscattering energy profiles, demonstrating that extinction coefficients vary under different polarization modes [10].

Another approach to studying vegetation microwave scattering characteristics is the polarization decomposition method based on the target polarization scattering matrix. Polarization decomposition algorithms are mainly divided into two categories: eigenvalue decomposition-based algorithms and model decomposition-based algorithms, with the latter being widely applied in forest scattering characteristics analysis due to the physical interpretability of the decomposition results [11]. In 1998, Freeman and Durden abstracted the scattering characteristics of forests into three types, surface scattering, double-bounce scattering, and volume scattering, and proposed a three-component decomposition model [12]. Each scattering component of this model has clear physical properties, providing a new idea for the study of scattering characteristics. In 2007, Freeman proposed a two-component decomposition model for forests based on the three-component model and conducted experiments on tropical rainforests and temperate forests, finding that scattering characteristics were related to canopy closure and crown morphology, although this relationship required further clarification [13]. In recent years, a series of improved models that take into account the physical meaning of SAR signals have been proposed to represent the volume scattering energy more accurately. In 2010, An et al. addressed the issues of overestimating volume scattering energy and negative power in the Freeman–Durden model by adopting de-orientation, a new volume scattering model, and power constraints to improve the three-component decomposition algorithm [14]. In 2020, Wang et al. proposed a four-component decomposition method based on backscattering energy balance, which improved the accuracy of the volume scattering model, but the response relationship between the volume scattering component and the forest stand structural factors was still unclear [15].

A large number of microwave scattering measurements and polarization decomposition studies have been devoted to analyzing forest scattering characteristics. However, existing scattering measurement studies do not clearly demonstrate how variations in forest canopy structure affect backscattered energy across different frequency bands. There is a lack of comparative analysis between scattering measurement results and scattering characteristics obtained from model decomposition. The correspondence between scattering characteristics obtained from polarization decomposition algorithms and stand structure remains unclear.

In this study, we conducted multi-angle full-polarization scattering measurements of Masson pine canopy scenes with different structures in C-, X-, and Ku-bands, combined with backscattering profile extraction and model-based polarization decomposition, to explore the scattering characteristics of Masson pine canopies and the sources of backscattering energy contribution. Our findings are expected to provide actionable insights for monitoring Masson pine growth and health using SAR data, improving the accuracy and reliability of forest parameter estimation.

## 2. Materials and Methods

### 2.1. Experimental Platform

**Figure 1:**
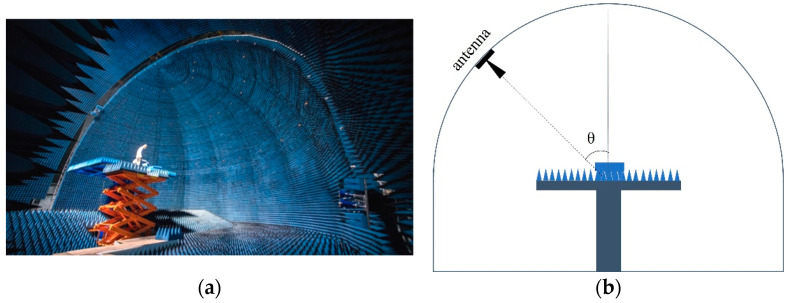
(**a**) Interior view of microwave characteristic measurement and simulation imaging science experiment platform (LAMP, Deqing, China); (**b**) Geometric diagram of the platform.

This study was carried out on the microwave characteristic measurement and simulation imaging science experiment platform of the laboratory of target microwave properties (LAMP). The platform, located in Deqing County, Zhejiang Province, China, provides a dome-shaped, interference-free microwave anechoic chamber for measurements. It can emit continuous spectra within the frequency range of 0.8–20 GHz and perform full-polarization target scattering measurements at incidence angles from 0–90° and azimuth angles from 0–360° [16]. The platform has specific dimensions of 24 m (length) × 24 m (width) × 17 m (height), with high-performance absorbing materials covering the dome interior, the exterior of the turntable, and the ground. The interior view of the microwave characteristic measurement and simulation imaging science experiment platform and the measurement indicators are shown in Figure 1 and Table 1, respectively.

### 2.2. Measurement Experiment Design

The measurement experiment design includes the acquisition of observation trees, scene construction, and configuration of measurement parameters.

**Figure 2:**
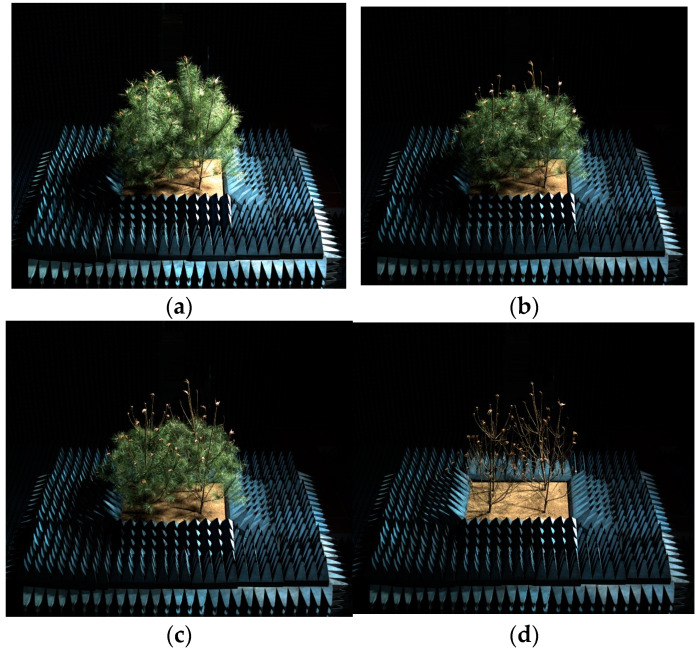
(**a**) The scene with all needles (S1), (**b**) the first defoliation scene (S2), (**c**) the second defoliation scene (S3), (**d**) the scene without needles (S4).

The observation samples in this study consisted of four three-year-old Masson pine trees, the largest size that the measurement platform could accommodate, all obtained from a nearby forest (30.5886° N, 199.9923° E). The average diameter at breast height (DBH) of the four young trees was 3.4 cm, and the average height was 2.0 m. The observation scene was constructed using a specialized container measuring 1.6 m (length) × 1.2 m (width) × 0.3 m (height), filled with soil obtained from the forest, with the soil height level with the container height. The four trees were planted in the center of the container in a 2 × 2 arrangement. After planting, the four trees formed a simple stand scene, with an average height of 1.8 m, a canopy thickness of 1.6 m, and both east-west and north-south canopy lengths of 2.0 m. The completed scene was placed at the center of the turntable, with absorbing material positioned outside the container to minimize external interference. This study included four observation scenes: the first was a scene with all needles (S1), the second was a scene where all needles on the first round of branches were removed (S2), the third was a scene where all needles on the first and second rounds of branches were removed (S3), and the fourth was a scene where all needles were removed (S4). Photos of each observation scene are shown in Figure 2.

In terms of measurement parameter settings, backscattering data were collected for each scene in C-, X-, and Ku-bands. The center frequencies for these bands were 5.40 GHz, 9.65 GHz, and 14.00 GHz, respectively, with a frequency sweep width of 1.4 GHz and a frequency point interval of 0.005 GHz, resulting in a total of 281 frequency point data for each band. The observed incidence angles ranged from 30° to 60° with an interval of 5° and included an additional observation at 0°. The observed azimuth angles ranged from 0° to 315° with an interval of 45°.

### 2.3. Data Processing

**Figure 3:**
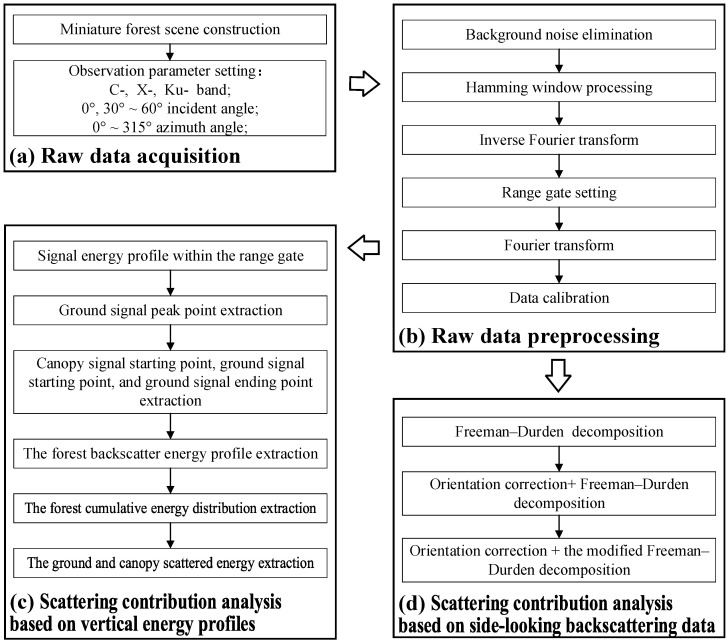
Workflow of this study.

Data processing mainly consisted of three parts (Figure 3). The first part was the preprocessing of backscattering measurement data, which involved a series of steps, including background noise elimination, Hamming window processing, inverse Fourier transform, range gate setting, Fourier transform, and data calibration, resulting in accurate backscattering complex data for each scene. The second part was scattering characteristics analysis based on vertical energy profiles. The analysis used data with 0° incidence angles to extract the starting point of the canopy signal, the peak point of the ground signal, and the termination point of the ground signal within the range gate, allowing for the derivation of the cumulative energy curve and the proportion of ground and canopy scattering energy of the scene. The final part was scattering characteristics analysis based on side-looking backscattering data using observations at incidence angles of 30° to 60°. The analysis performed polarization decomposition using the Freeman–Durden three-component model and a modified algorithm on the backscattering complex data to investigate the scattering characteristics of Masson pine scenes.

#### 2.3.1. Preprocessing on Backscattering Measurement Data

The raw data obtained from the measurement platform consists of amplitude and phase information recorded at discrete frequency points within the measurement frequency range, referred to as frequency domain data. To accurately acquire the target backscattering information, a series of preprocessing steps are required on the raw frequency domain data [17].

Background noise elimination was achieved by separately collecting backscattering data from the target scene and the empty scene. By performing complex subtraction on the two sets of obtained raw frequency domain data, background noise was removed from the backscattering data of the target scene. To minimize the effects during the periodic processing of the data, Hamming window processing was applied to the data prior to the inverse Fourier transformation. The inverse Fourier transformation converted frequency domain data into time domain data and then obtained the spatial domain data according to the propagation speed of the electromagnetic wave, which represented the spatial distribution of backscattering energy with respect to propagation distance in the indoor measurement environment. According to the relationship between the sweep frequency bandwidth during measurement and the distance resolution of the spatial domain data (Equation (1)), the sweep frequency bandwidth for all three observed bands in this study was 1.4 GHz, resulting in a distance resolution of 0.11 m for the spatial domain data. Further, based on the position and dimensions of the target object, the specific range of the target’s backscattering contribution was determined. By setting a range gate, the background noise outside the target’s backscattering range was effectively eliminated. The data within the range gate represented the backscattering energy profile of the target object along the observation path. Considering the dimensions of the observed forest scene and various observation angles, this study set the range gate to ±1.5 m. Finally, the spatial domain data were transformed back into frequency domain data using Fourier transformation, allowing for the acquisition of the complex backscattering data of the target at each frequency point within the measurement frequency range. In addition, this study employed the three-target polarization calibration algorithm with a metal disk, a dihedral angle of 90°, and a dihedral angle of 45° as standard calibrator to calibrate the observation data [18], as follows:

(1) $$d=\frac{c}{2B}$$

where d is the distance resolution of the microwave signal, c is the speed of light, and B is the sweep width.

#### 2.3.2. Analysis Based on Vertical Energy Profiles

To clarify the relationship between the backscattering energy and the scatterers of the scene, this study selected the backscattering energy profile at the nadir direction (0° incidence angle) for scattering contribution analysis.

**Figure 4:**
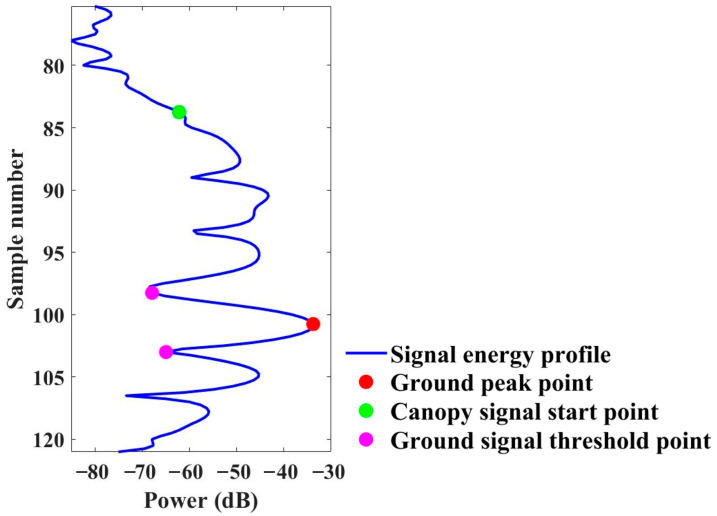
Illustration of backscatter energy profile and signal locations of canopy and ground.

The backscattering energy profiles were extracted within the range gate. For each energy profile, the position of the ground and the top of the canopy in the energy profile can be automatically identified by energy mutation detection [19], as shown in Figure 4. Specifically, the process began by manually determining the potential range for the occurrence of the ground peak. Then, within this potential range, the location of the maximum signal intensity was identified as the position of the ground signal peak. The starting point of the canopy signal was determined based on the position of the ground signal peak and the measured height of the forest scene. The starting and ending points of the ground signal were defined through energy changes near the ground signal peak. By performing a second derivative analysis on the energy profile, the maximum points were calculated on the second derivative curve of the original signal. The first maximum points on either side of the ground signal peak defined the starting and ending points of the ground signal.

In this study, the observed energy profiles of eight azimuths in the vertical observation direction were averaged to obtain the backscattered energy profiles of the scene for the energy contribution analysis. Firstly, based on each energy position point, the scattering energy ratio between the canopy and the ground of each energy profile was calculated, and the overall scattering energy contribution law of the Masson pine stand was analyzed. Secondly, based on the energy profiles, the cumulative energy distribution curves were calculated, and the scattering energy contribution rule of the branches and leaves was analyzed.

#### 2.3.3. Analysis Based on Side-Looking Backscattering Data

To explore the relationship between microwave signals obtained in side-looking mode and forest canopy structure, this study analyzed the variation of backscattering energy across different bands, polarization modes, and incident angles for various Masson pine canopy structures. Then, polarization decomposition was conducted using the Freeman–Durden model, the Freeman–Durden model combined with orientation correction, and the modified Freeman–Durden model combined with orientation correction, respectively. The energy distribution of each polarization characteristic was analyzed across various scenes, bands, observation angles, and decomposition algorithms. The meaning of the forest stand structure of polarization characteristics in each band was explored.

The backscattering energy of the observation results was calculated by the backscattering complex data at the center frequency point. The model-based polarization decomposition algorithm requires the average polarization coherence matrix of the target. This study constructed the target polarization scattering matrix (Equation (2)) through the backscattered complex data of four polarization modes, then decomposed the S matrix through the Pauli basis and constructed the polarization coherence matrix (Equation (3)). Finally, the average polarization coherence matrix was calculated using the polarization coherence matrices obtained under eight observation azimuths, as follows:

(2) $$S=\left[\begin{array}{cc} S_{hh} & S_{hv}\\ S_{vh} & S_{vv} \end{array}\right]$$

where S is the polarization scattering matrix, $S_{ij}$ is the backscattering complex data in ij mode, i is the polarization mode of the received electromagnetic wave, and j is the polarization mode of the transmitted electromagnetic wave; when i and j are same, it is called co-polarization, and when i and j are different, it is called cross-polarization.

(3) $$T=\frac{1}{2}\left[\begin{array}{ccc} \langle {\left|S_{hh}+S_{vv}\right|}^{2}\rangle & \langle \left(S_{hh}+S_{vv}\right){\left(S_{hh}-S_{vv}\right)}^{*}\rangle & 2\langle \left(S_{hh}+S_{vv}\right){S_{hv}}^{*}\rangle \\ \langle \left(S_{hh}-S_{vv}\right){\left(S_{hh}+S_{vv}\right)}^{*}\rangle & \langle {\left|S_{hh}-S_{vv}\right|}^{2}\rangle & 2\langle \left(S_{hh}-S_{vv}\right){S_{hv}}^{*}\rangle \\ 2\langle S_{hv}{\left(S_{hh}+S_{vv}\right)}^{*}\rangle & 2\langle S_{hv}{\left(S_{hh}-S_{vv}\right)}^{*}\rangle & 4\langle {\left|S_{hv}\right|}^{2}\rangle \end{array}\right]$$

where $\langle A\rangle$ is the arithmetic mean of A, $\left|A\right|$ is the modulus of A, and A* is the conjugate of A.

Based on the average polarization coherence matrix, model-based polarization decomposition algorithms were employed to calculate the polarization characteristics of the scenes. The first approach utilized the Freeman–Durden model, which decomposed the average polarization coherence matrix into surface scattering, double-bounce scattering, and volume scattering (Equation (4)), as follows:

(4) $$T=P_{s}T_{s}+P_{d}T_{d}+P_{v}T_{v}$$

where $P_{s}$, $P_{d}$, $P_{v}$ represents the power of surface scattering, double-bounce scattering, and volume scattering respectively, $T_{s}$, $T_{d}$, $T_{v}$ represents the model of surface scattering, double-bounce scattering, and volume scattering respectively.

The second approach was the polarization decomposition using the Freeman–Durden model combined with orientation correction. The orientation angle refers to the angle of rotation of the target in the plane perpendicular to the radar’s incident direction. It is widely present in scattering data and affects the analysis of the target’s scattering characteristics. Orientation correction is achieved by rotating the target’s orientation angle to a standardized position, thereby eliminating the effects of randomly distributed orientation angles [20]. The average polarization coherence matrix was calculated based on the corrected polarization scattering matrix, and then the Freeman–Durden model was used to extract the polarization characteristics of the scene, as follows:

(5) $$S_{0}=\left[\begin{array}{cc} \cos\theta & \sin\theta \\ -\sin\theta & \cos\theta \end{array}\right]S\left[\begin{array}{cc} \cos\theta & -\sin\theta \\ \sin\theta & \cos\theta \end{array}\right]$$

where S is the matrix containing the orientation angle, $S_{0}$ is the matrix rotated to the standard position, and θ is the orientation angle.

The third approach utilized the modified Freeman–Durden model combined with orientation correction. Given that the Freeman–Durden model tends to overestimate volume scattering energy and result in negative energy for other components [21], An et al. proposed a volume scattering model with polarization entropy equal to one (Equation (6)), which modified the Freeman–Durden model and addressed the estimation issues [14], as follows:

(6) $$T_{vol}=\frac{1}{3}\left[\begin{array}{ccc} 1 & 0 & 0\\ 0 & 1 & 0\\ 0 & 0 & 1 \end{array}\right]$$

where $T_{vol}$ is the new volume scattering model proposed by An et al. [14] to replace the volume scattering part of the Freeman–Durden model.

## 3. Results

### 3.1. Scattering Characteristics Analysis Based on Vertical Energy Profiles

**Figure 5:**
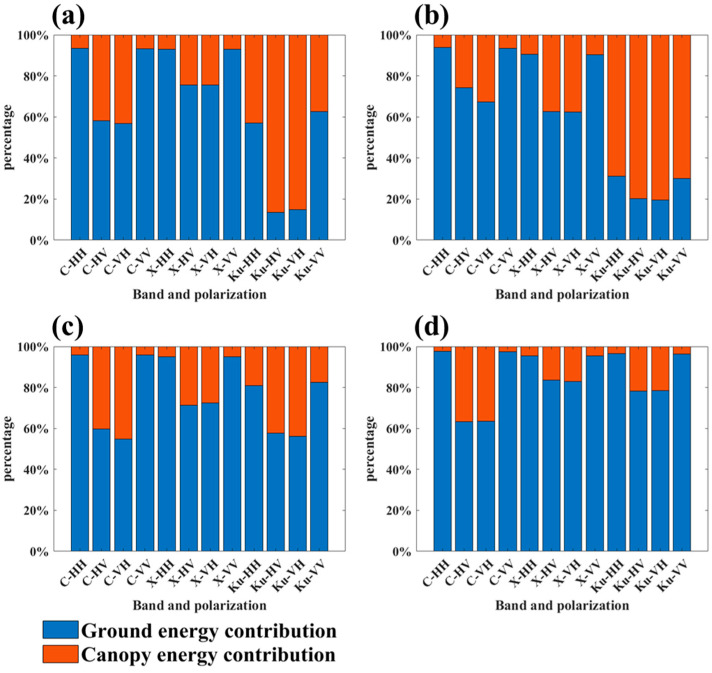
Statistics of the ground and canopy energy contribution ratios for different canopy structure scenes: (**a**) scene S1; (**b**) scene S2; (**c**) scene S3; (**d**) scene S4.

Vertical observation of backscattering energy can intuitively reflect the sources of energy contribution. The statistical results of ground and canopy energy contributions for full polarization modes in the C-, X-, and Ku-bands for different canopy structure scenes are shown in Figure 5.

As shown in Figure 5, for the complete Masson pine canopy structure, all polarization modes in the C- and X-bands, as well as the co-polarization mode in the Ku-band, exhibited that ground energy contribution constituted the major part of the total backscattering energy. The cross-polarization mode in the Ku-band showed that canopy scattering contributed the majority of the total backscattering energy. Specifically, the ground scattering energy contribution ratios for co- and cross-polarization modes were 93% and 58% in the C-band, respectively. In the X-band, the ground contribution ratios were 93% for co-polarization and 75% for cross-polarization. In the Ku-band, the ratios dropped to 60% for co-polarization and 14% for cross-polarization. Additionally, the canopy scattering energy contribution ratio for cross-polarization was consistently higher than that for co-polarization across all three bands.

Throughout the defoliation process of the Masson pine canopy, variations in backscattering energy contributions were observed across different bands and polarization modes. As defoliation progresses, the ground energy contribution in the C-band for co- and cross-polarization modes showed a slight increase, rising from 93% to 98% and from 58% to 63%, respectively. Similarly, in the X-band, ground energy contributions for co- and cross-polarization modes increased modestly, from 93% to 95% and from 75% to 83%, respectively. In contrast, the Ku-band demonstrated more substantial changes: with the reduction in canopy needles, the ground energy contribution in the co- and cross-polarization modes rose sharply, from 60% to 96% and from 14% to 78%, respectively.

It is worth noting that during the defoliation process of the Masson pine canopy, changes in canopy energy contributions exhibited non-monotonic trends in certain bands and polarization modes. In the cross-polarization modes of the X-bands, the proportion of canopy scattering energy unexpectedly increased after the first defoliation. Similarly, in the Ku-band co-polarization mode, canopy scattering energy also showed an increase following the first defoliation.

**Figure 6:**
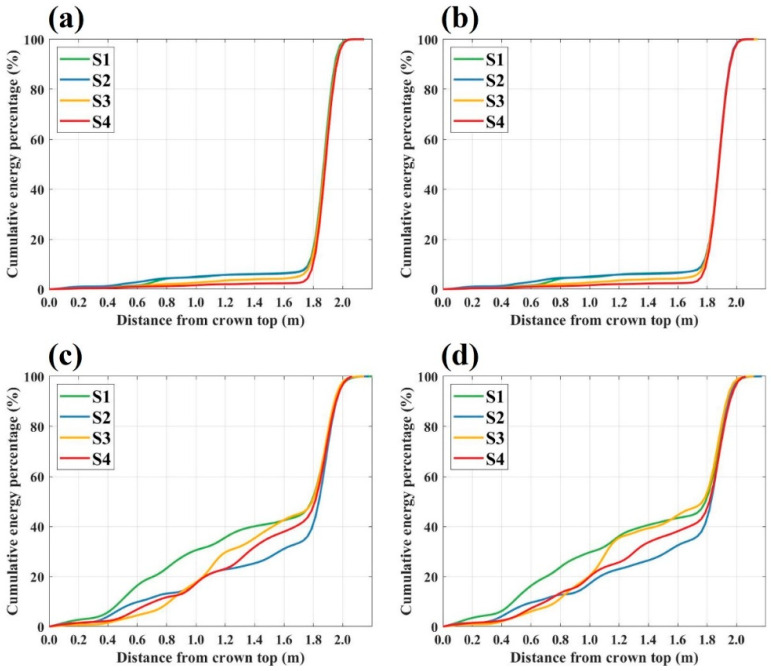
Cumulative backscattering energy distribution curves for various scenes under different polarization modes in the C-Band: (**a**) HH polarization mode; (**b**) VV polarization mode; (**c**) HV polarization mode; (**d**) VH polarization mode.

**Figure 7:**
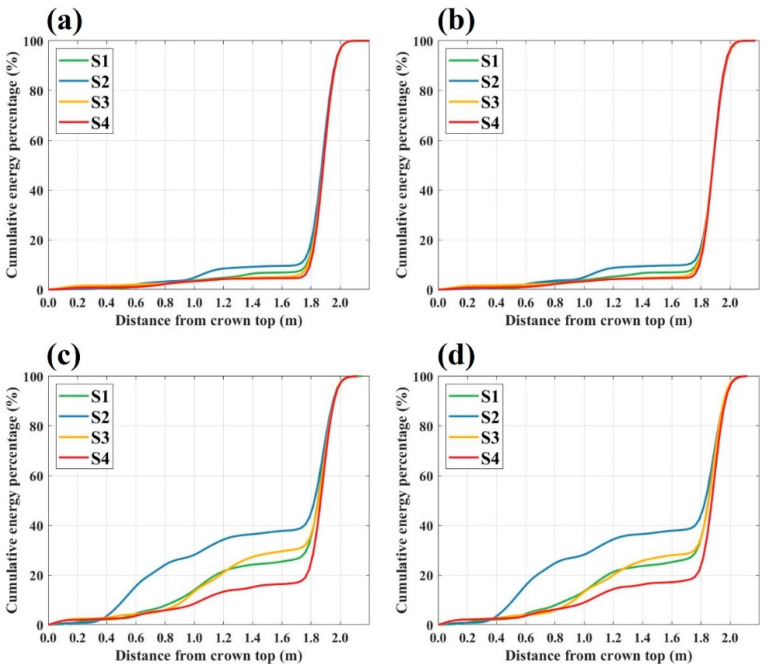
Cumulative backscattering energy distribution curves for various scenes under different polarization modes in the X-Band: (**a**) HH polarization mode; (**b**) VV polarization mode; (**c**) HV polarization mode; (**d**) VH polarization mode.

**Figure 8:**
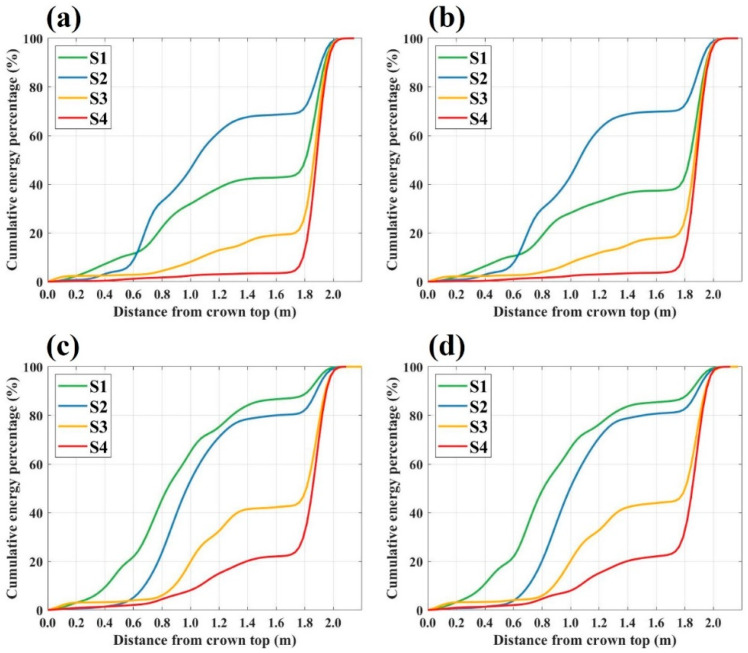
Cumulative backscattering energy distribution curves for various scenes under different polarization modes in the Ku-Band: (**a**) HH polarization mode; (**b**) VV polarization mode; (**c**) HV polarization mode; (**d**) VH polarization mode.

To further investigate the microwave scattering characteristics of the Masson pine in response to canopy defoliation, cumulative backscattering energy distribution curves were generated for the four canopy structure scenes across all polarization modes in the C-, X-, and Ku-bands, as presented in Figure 6, Figure 7 and Figure 8.

Figure 6 illustrates the cumulative backscattering energy distribution curves for different canopy structure scenes under various polarization modes in the C-band. For the co-polarization mode, backscattering energy was predominantly concentrated at the ground level, specifically beyond 1.8 m from the canopy top, with less than 10% of the total backscattering energy originating from the canopy itself. Differences in energy distribution across canopy structure scenes were relatively minor, with the scenes S1 and S2 exhibiting the highest and similar backscattering energy levels, followed by scenes S3 and S4, showing the relatively low backscattering energy among all scenes.

In the cross-polarization mode of the C-band, canopy backscattering energy contributed up to 50% of the total backscattering energy, with notable variations across different canopy structure scenes. In the scene with full needles, backscattering energy remained the highest across all heights. Among the three defoliation scenes, before reaching 1.0 m from the canopy top, the backscattering energy ranked from high to low as follows: scene S2, scene S4, and scene S3. From 1.0 m down to the ground, this order shifted, with backscattering energy ranking from high to low as follows: scene S3, scene S4, and scene S2.

Figure 7 presents the cumulative backscattering energy distribution curves in the X-band. In the co-polarization mode, backscattering energy was primarily concentrated at the ground level, beyond 1.8 m from the canopy top, with about 10% of the total energy originating from the canopy region. Energy differences among the canopy structure scenes were minimal, particularly in the upper canopy, where backscattering energy distribution remained largely consistent across all scenes. However, in the lower canopy region, scene S2 demonstrated relatively higher energy levels, whereas scenes S1, S3, and S4 exhibited lower energy.

In the cross-polarization mode of the X-band, the canopy backscattering energy contribution ranged from 22% to 43%. Up to 0.4 m from the canopy top, the energy distribution remained consistent across all scenes. Beyond this point, scene S2 displayed a higher energy distribution, while scenes S1 and S3 exhibited similar energy levels, and scene S4 showed the lowest energy levels.

Figure 8 illustrated the cumulative backscattering energy distribution curves within the Ku-band. In the co-polarization mode, the cumulative canopy energy distribution varied significantly across scenes, ranging from 10% to 70%, highlighting a strong dependency on canopy structure. Specifically, in the region up to 0.6 m from the canopy top, scene S1 showed the highest energy intensity, while the other scenes displayed similar and lower energy intensities. Beyond 0.6 m, the energy intensity in scene S2 surpassed that in scene S1, with cumulative canopy energy reaching 70%, compared to 47% in scene S1. Scene S3 ranked third, achieving a cumulative canopy energy of 24%, while scene S4 showed the lowest canopy energy, with a cumulative value of 10%.

In the cross-polarization mode of the Ku-band, cumulative canopy energy contributions varied significantly across different canopy structure scenes, ranging from 29% to 88%, indicating a strong dependency on canopy structure. Scene S1 consistently exhibited the highest canopy energy at all heights. In scene S2, canopy energy began to increase from 0.6 m below the canopy top, reaching a cumulative energy of 81% before ground level, second only to scene S1. For scenes S3 and S4, cumulative canopy energy contributions before reaching the ground were 46% and 29%, respectively, reflecting comparatively lower energy contributions from the canopy.

### 3.2. Scattering Characteristics Analysis Based on Side-Looking Backscattering Data

#### 3.2.1. Backscattering Energy Intensity in Masson Pine Canopy

**Figure 9:**
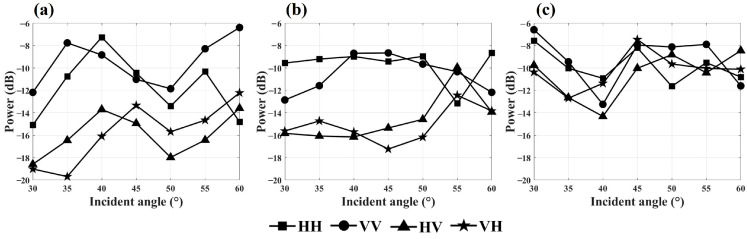
Variation of backscattering energy with observation incidence angle for scene S1: (**a**) in the C-band; (**b**) in the X-band; (**c**) in the Ku-band.

To investigate the impact of observation angles on the backscattering energy of the Masson pine canopy, variations in backscattering energy relative to the observation incidence angle were plotted across four polarization modes in the C-, X-, and Ku-bands, based on scene S1 (Figure 9).

As illustrated in Figure 9, the backscattering energy distribution for the co-polarization and cross-polarization modes in the C-band ranged from −15 dB to −6 dB and −20 dB to −12 dB, respectively, with standard deviations of 2.5 dB and 2.4 dB. In the X-band, the backscattering energy in the co-polarization and cross-polarization modes ranged from −13 dB to −9 dB and −17 dB to −10 dB, with standard deviations of 1.6 dB and 1.9 dB, respectively. For the Ku-band, the ranges of the backscattering energy were −12 dB to −7 dB for co-polarization and −14 dB to −7 dB for cross-polarization, with both modes showing a standard deviation of 1.9 dB.

The canopy backscattering energy was influenced by the observation incidence angle. Specifically, cross-polarization backscattering energy increased with larger observation incidence angles, while co-polarization backscattering energy did not exhibit a clear trend with angle changes. Among the bands, the Ku-band showed the highest backscattering energy, followed by the X-band, with the C-band displaying the lowest energy. Additionally, the C-band demonstrated the highest sensitivity to variations in the observation incidence angle.

For polarization modes, co-polarization generally yielded higher backscattering energy than cross-polarization. Notably, in the Ku-band, cross-polarization backscattering energy was sensitive to the Masson pine needles, with values approaching those in the co-polarization mode.

**Figure 10:**
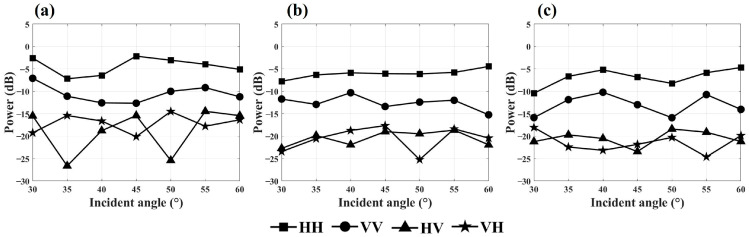
Variation of backscattering energy with observation incidence angle for scene S1 after de-orientation: (**a**) in the C-band; (**b**) in the X-band; (**c**) in the Ku-band.

Orientation correction mitigated the influence of orientation angles on the target’s backscattering energy. Applying orientation correction to the polarization scattering matrix, the variations in backscattering energy relative to the observation incidence angle for scene S1 across the four polarization modes in the C-, X-, and Ku-bands are illustrated in Figure 10.

As shown in Figure 10, orientation correction reduced the impact of observation incidence angle on backscattering energy. After the correction, the variation in backscattering energy across different incidence angles decreased in the co-polarization mode, while it increased in the cross-polarization mode. For the C-band, the standard deviation of backscattering energy across incidence angles after de-orientation was 2.0 dB for co-polarization and 3.6 dB for cross-polarization. In the X-band, these standard deviations were 1.2 dB for co-polarization and 2.2 dB for cross-polarization. For the Ku-band, post-de-orientation standard deviations were 2.1 dB for co-polarization and 1.9 dB for cross-polarization.

**Figure 11:**
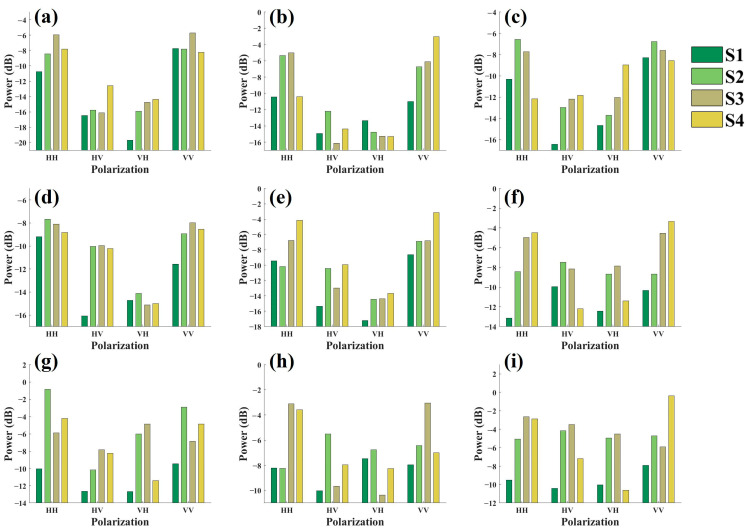
Side-looking backscattering energy for different canopy structure scenes of Masson pine: (**a**–**c**) represents the backscattering energy of the C-band at incidence angles of 35°, 45°, and 55°, respectively; (**d**–**f**) represents that of the X-band at incidence angles of 35°, 45°, and 55°, respectively; (**g**–**i**) represents that of the Ku-band at incidence angles of 35°, 45°, and 55°, respectively.

To investigate the effect of canopy structure on the side-looking backscattering energy, backscattering energy distributions were plotted for four canopy scenes across three frequency bands, four polarization modes, and incidence angles of 35°, 45°, and 55°, as illustrated in Figure 11.

As illustrated in Figure 11, the defoliation process in the Masson pine scenes altered the original backscattering energy distribution, with distinct variation patterns observed across different bands, polarization modes, and incidence angles. Scene S1 typically exhibited lower backscattering energy than other defoliated scenes across all observation modes, and as defoliation progressed, the backscattering energy of the scene generally increased. However, the scene exhibiting the maximum backscattering energy varied depending on the observation mode, with the peak appearing in scene S2, S3, or S4.

**Figure 12:**
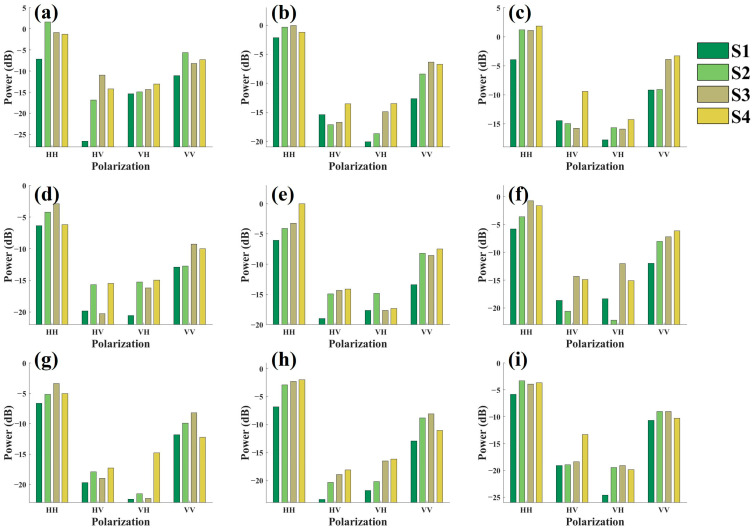
Side-looking backscattering energy for different canopy structure scenes of Masson pine after orientation correction: (**a**–**c**) represents the backscattering energy of the C-band at incidence angles of 35°, 45°, and 55°, respectively; (**d**–**f**) represents that of the X-band at incidence angles of 35°, 45°, and 55°, respectively; (**g**–**i**) represents that of the Ku-band at incidence angles of 35°, 45°, and 55°, respectively.

For the polarization scattering matrix after orientation correction, the side-looking backscattering energy for each canopy structure scene was plotted (Figure 12). The consistency in backscattering energy variation across different canopy structures improved. As canopy needles decreased, each scene exhibited a gradual increase in backscattering energy. In most observation modes, the maximum backscattering energy was achieved either after the second defoliation or in the completely leafless scene.

This analysis suggested that backscattering energy in the Masson pine scene predominantly originated from ground contributions, with the canopy primarily acting to attenuate the microwave signal. Consequently, this attenuation effect made it challenging to use microwave backscattering energy directly for parameter inversion of Masson pine canopy characteristics.

#### 3.2.2. Polarization Characteristic Energy Variation in Masson Pine Canopy

To investigate the relationship between Masson pine canopy structure and microwave signal response, polarization decomposition was applied to observational data from various scenes using model-based decomposition. Given that there were errors in the decomposition results of polarization characteristics, this study evaluated the energy errors of different polarization decomposition results based on the principle of energy conservation before and after decomposition.

**Figure 13:**
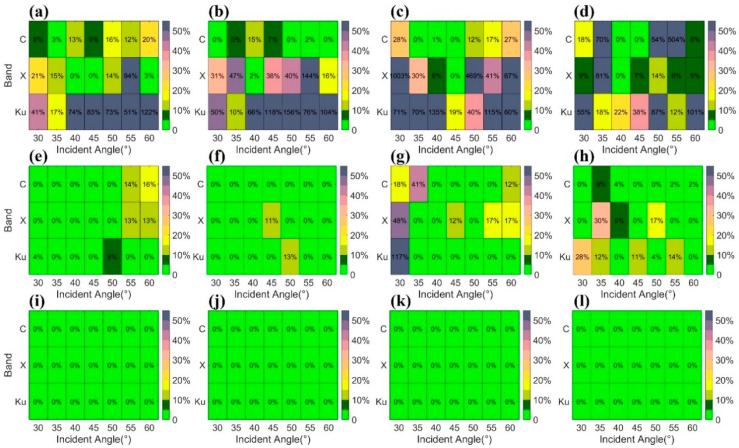
Decomposition energy error statistics based on different polarization decomposition algorithms: (**a**–**d**) represents the energy error distribution under different incident angles for scenes S1, S2, S3, and S4, respectively, using the Freeman–Durden model decomposition; (**e**–**h**) represents that for scenes S1, S2, S3, and S4, respectively, using the decomposition based on the Freeman–Durden model combined with orientation correction; (**i**–**l**) represents that for scenes S1, S2, S3, and S4, respectively, using the decomposition based on the modified Freeman–Durden model combined with orientation correction.

Figure 13 presented the decomposition energy errors for three polarization decomposition algorithms: the Freeman–Durden model, the Freeman–Durden model combined with orientation correction, and the modified Freeman–Durden model combined with orientation correction. The results indicated that the decomposition based on the Freeman–Durden model alone exhibited significant decomposition energy errors, particularly in the X-band and Ku-band, with average decomposition energy errors of 30% for the C-band, 79% for the X-band, and 67% for the Ku-band.

Applying orientation correction to the polarization scattering matrix significantly reduced decomposition energy errors for the Freeman–Durden model. The average decomposition energy errors dropped to 4% for the C-band, 7% for the X-band, and 8% for the Ku-band. Furthermore, the combination of the modified Freeman–Durden model and orientation correction eliminated decomposition energy errors entirely, achieving zero energy error across all bands.

Polarization decomposition was conducted on observational data for four canopy structure scenes using the three polarization decomposition algorithms. The data comprised observations at C-, X-, and Ku-bands, with incidence angles of 35°, 45°, and 55°. For each scene and band, the surface scattering, double-bounce scattering, and volume scattering energy contributions were calculated to analyze the scattering characteristics of the Masson pine canopy in side-looking mode.

**Figure 14:**
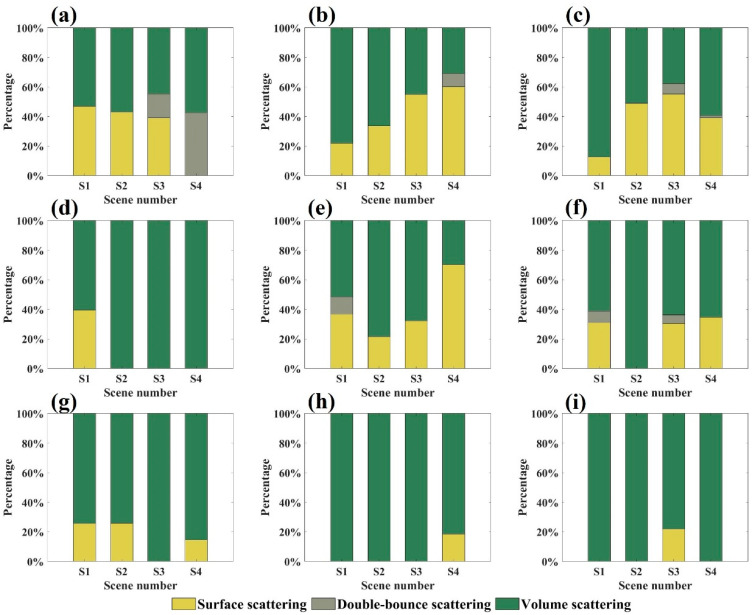
The scattering characteristics energy proportion of each scene obtained by the Freeman–Durden model: (**a**–**c**) represents the energy proportion of the C-band at incidence angles of 35°, 45°, and 55°, respectively; (**d**–**f**) represents that of the X-band at incidence angles of 35°, 45°, and 55°, respectively; (**g**–**i**) represents that of the Ku-band at incidence angles of 35°, 45°, and 55°, respectively.

Figure 14 illustrates the scattering characteristic contributions obtained using the Freeman–Durden model-based polarization decomposition algorithm. Across all scenes, volume scattering emerged as the dominant contributor, with contributions reaching up to 100% in certain cases, especially for the X- and Ku-bands. Surface and double-bounce scattering contributions were markedly lower in all scenes. Combined with the large error in the decomposition energy of this algorithm, this result clearly showed that the decomposition result of the Freeman–Durden model had the defects of overestimation of volume scattering energy and negative power in surface scattering and double-bounce scattering.

**Figure 15:**
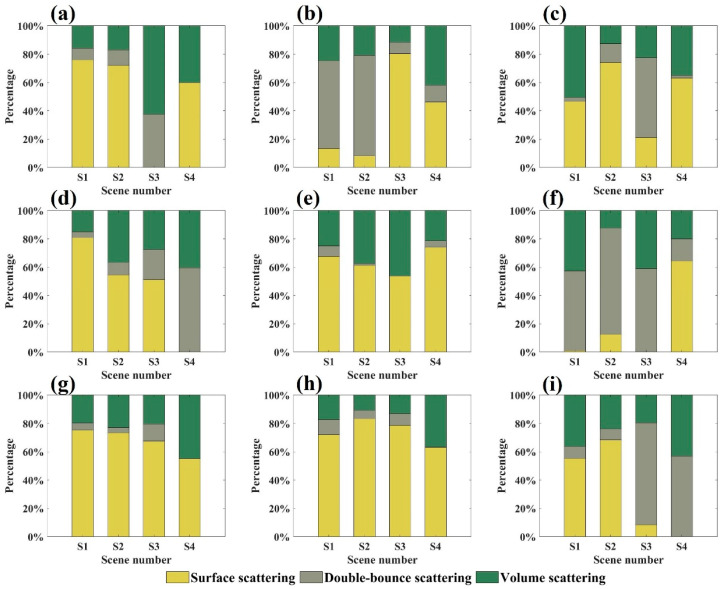
The scattering characteristics energy proportion of each scene obtained by the modified Freeman–Durden model combined with orientation correction: (**a**–**c**) represents the energy proportion of the C-band at incidence angles of 35°, 45°, and 55°, respectively; (**d**–**f**) represents that of the X-band at incidence angles of 35°, 45°, and 55°, respectively; (**g**–**i**) represents that of the Ku-band at incidence angles of 35°, 45°, and 55°, respectively.

Figure 15 presents the scattering characteristic contributions derived from the decomposition based on the modified Freeman–Durden model combined with orientation correction. The shortcomings observed in the traditional Freeman–Durden model have been effectively addressed through the combination algorithm. Scattering contributions display variability across different bands and canopy structure scenes, reflecting a more accurate representation of the physical scattering processes.

For the C-band, the average volume scattering contribution ranged from 17% to 39%, the average surface scattering contribution ranged from 34% to 56%, and the average double-bounce scattering contribution ranged from 5% to 34%. For the X-band, the average volume scattering contribution ranged from 27% to 38%, the average surface scattering contribution ranged from 35% to 50%, and the average double-bounce scattering contribution ranged from 23% to 28%. For the Ku-band, the average volume scattering contribution ranged from 18% to 41%, the average surface scattering contribution ranged from 39% to 75%, and the average double-bounce scattering contribution ranged from 5% to 31%.

As the needles of the Masson pine canopy were gradually removed, the proportion of volume scattering energy in each scene changed differently. For example, the maximum proportion of volume scattering energy in the C-band observation scenes with incident angles of 35°, 45°, and 55° appeared in scene S3, S4, and S1, respectively, while the maximum proportion of volume scattering energy in the Ku-band appeared in scene S4.

## 4. Discussion

### 4.1. Analysis of Ground and Canopy Scattering Contribution Based on Vertical Energy Profiles

This study calculated vertical backscattered energy profiles for each canopy structure scene, band, and polarization mode to investigate the ground and canopy scattering contribution ratios in Masson pine forests.

Across all bands, ground energy generally dominated the total backscattering energy in co-polarization modes. This trend was particularly pronounced in the C- and X-bands, where the ground energy contribution remained high (93% for co-polarization in both bands) and showed moderate variation with canopy defoliation. In contrast, the Ku-band demonstrated more variability and sensitivity to canopy structure, with ground energy contributions significantly influenced by polarization mode. For instance, while the Ku-band co-polarization mode showed a ground contribution of 60%, the ground energy contribution sharply dropped to 14% in the cross-polarization, highlighting a stronger canopy signal. This suggests that higher-frequency bands, like the Ku-band, could capture more detailed canopy information, which is potentially useful for detailed canopy structure analysis. Similar results appeared in the data analysis and radiation transfer simulation based on UAV profile radar. This research employed the X-band UAV profile radar system, HUTSCAT, to conduct a correlation analysis between the backscattering profiles and the forest parameters [22], and a radiative transfer model, MIT/CESBIO, was developed to simulate the backscatter profiles for penetration depth analysis [23]. The results indicated that the ground backscattering accounted for the majority in forest stands with a lower branch density. The Ku-band profiles and radiative transfer simulation results indicated that the Ku-band signal contained rich canopy scattering information, and the canopy scattering energy contribution in cross-polarization was higher than that in co-polarization [19,24,25].

### 4.2. Analysis of Branches and Needles Scattering Contributions Based on Vertical Energy Profiles

The defoliation process combined with the vertical cumulative energy distribution helps to explore the scattering contributions of canopy branches and needles in detail. According to the cumulative energy distribution in the C-band co-polarization mode (Figure 6a,b), the top branches and needles had almost no response to the signal, while that in the middle and lower parts of the canopy contributed about 10% of the scattered energy. In view of the phenomenon that the higher the density of canopy needles, the stronger the canopy backscattering energy, the branches and needles of the Masson pine canopy mainly caused scattering in the C-band co-polarization mode, while the extinction effect was relatively weak. By comparing the canopy cumulative energy distribution in the scene with full needles and without needles, the Masson pine branches contributed 7.7% of the backscattering energy, and the needles contributed 5.5%. For the C-band cross-polarization mode (Figure 6c,d), the signal was scattered and attenuated in the canopy, which was evidenced by the difference in energy changes between the upper and lower parts of the canopy during defoliation. From the measurement results of the scene without needles, it could be obtained that the scattering contribution of branches was 46.3%, while, due to the effect of extinction, the scattering contribution of the scene with full needles was only 3.8% higher than that.

In the C-band co-polarization mode, the scene after the first defoliation exhibited the highest accumulation energy in the lower part of the canopy, reflecting the extinction effect of the upper needles on the signal, and the scattering contributions of branches and needles were 8.9% and 7.4%, respectively. In the X-band cross-polarization mode, the scene with the highest scattering energy in the middle and lower parts of the canopy was still the S2 scene, with the scattering contributions of branches and needles being 22.3% and 20.7%, respectively. In addition, based on the distribution range of backscattering energy, the co-polarization scattering contribution in the X-band mainly came from the larger branches and needles in the lower part of the canopy, while in the cross-polarization mode, the contribution came from both the middle and lower parts of the canopy.

In the Ku-band co-polarization mode (as shown in Figure 8a,b), the needles in the upper canopy can scatter the signal and have a strong extinction effect, so that the backscattering energy of the scene with full needles was higher than that of the first defoliation scene in the upper canopy but lower than that of the first defoliation scene in the lower canopy. Comparing the backscattering energy accumulation of different canopy structures, the branches contributed 9.9% of the scattering energy, while the needles contributed 60.7%. In the Ku-band cross-polarization mode, the branches and leaves of the Masson pine canopy mainly scattered the signal, which was reflected by the consistent trend of the canopy accumulated energy and the canopy needle density. The scattering energy contributions of branches and needles accounted for 29.5% and 58.8%, respectively, in the Ku-band cross-polarization mode. Based on the sensitivity of the Ku-band signals to the branches and needles of forest canopy, a UAV-mounted Ku-band profile radar has been developed and utilized in forest parameter estimation studies [25,26]. For example, by integrating forest growth models with radiation transfer models, the leaf area index (LAI) of the forest canopy and its uncertainty were successfully estimated using the Ku-band profiles [25].

### 4.3. Analysis of Energy Variations in Side-Looking Backscatter Data

This study investigated the effect of incidence angle on the side-looking backscattering energy of Masson pine scenes, as well as the role of orientation correction. The measurement results of the scene with full needles showed that the incident angle can affect the backscattering intensity of the scene. Similar results appeared in studies based on SAR data. Kovacs et al. analyzed the relationship between microwave signals and mangrove structural parameters based on Radarsat-2 C-band data and pointed out that the correlation between backscattering intensity and forest parameters decreased with increasing incident angle, while the correlation increased when the incident angle decreased [27]. By incorporating the incident angle information into the transfer learning model, Qin et al. improved the accuracy of crop classification using L-band UAV SAR data [28]. Additionally, data acquired at a specific incidence angle can capture target structure information along the direction. Therefore, combining multi-incident angle data can improve the detection ability of microwave data, such as the polarization tomography technology [29].

When using SAR data for forest parameter inversion, researchers try to reduce the impact of incidence angle on the inversion accuracy by using orientation correction. Zhang et al. increased the sensitivity and saturation of polarization characteristics to forest structural parameters and improved the inversion accuracy of forest aboveground biomass by applying orientation correction in L-band quad-polarimetric SAR images [20]. In this study, it is noteworthy that orientation correction can maximize the co-polarization energy of the target while reducing the standard deviation of co-polarized backscattering energy across different incidence angles. It effectively mitigated the influence of incidence angle on observational data, enhancing the consistency of the measurements. For the Freeman–Durden model decomposition algorithm, the polarization decomposition with the original polarization scattering matrix resulted in numerous overestimations of volume scattering and the occurrences of negative power. By using the corrected polarization scattering matrix for decomposition, the energy errors in each scattering characteristic were significantly reduced, which may help improve the accuracy of parameter inversion.

For scenes with different canopy structures, side-looking backscattering data were insufficient to accurately describe the canopy information. This study analyzed the backscattering energy across various canopy structure scenes. The observed low consistency between backscattering energy and canopy density may be attributed to the combined effects of the vegetation canopy and the ground. The interplay between scattering and attenuation within the canopy likely contributed to the variability, complicating the relationship between canopy density and backscattering energy. Additionally, the range ambiguity of microwave signals may also result in similar backscattering energy in different canopy structures because the target signal echoes are mixed with noise echoes. To solve this problem, the blind source separation algorithm has been proposed to suppress the range ambiguity of the microwave signal and highlight the target signal [30]. The model-based polarization decomposition algorithm seeks to improve the correlation between vegetation parameters and microwave signals by decomposing backscattering energy into distinct polarization components. However, this study found that even when the total energy of the polarization decomposition results matched the total energy before decomposition and no negative power values existed, the variations in volume scattering energy or other polarization components still failed to consistently align with changes in vegetation canopy structure. This phenomenon manifested in forest parameter inversion as a weak correlation between the various polarization components and the estimated parameters, resulting in a low coefficient of determination between the inversion results and the true values and a relatively large root mean square error [31,32]. Therefore, it is necessary to shift the focus from simply pursuing high correlation obtained by regression methods to accurately characterizing the interaction process between signals and scenes, thereby achieving more accurate and reliable inversion results.

## 5. Conclusions

This study investigated the multi-band microwave backscattering characteristics of Masson pine canopies with varying structures. Four scenes were constructed: a scene with all needles (S1), needles removed from the first round of branches (S2), needles removed from the first and second rounds of branches (S3), and all needles removed (S4). Based on the microwave anechoic chamber measurement platform, the backscattering data at C-, X- and Ku-bands were collected for each scene across eight incidence angles and eight azimuth angles to investigate the multi-band microwave backscattering characteristics of Masson pine stands. Specifically, the 0° incidence angle data were used to obtain the vertical backscattering energy profiles of the scene, from which the scattering energy contributions of the ground, crown, and the branches and needles of Masson pine in each band were quantified. The incidence angle data from 30° to 60° with a 5° step were used to collect the side-looking backscattering complex data. Based on the data, the effects of the incident angle and canopy structure on the backscattering energy in each band, as well as the role of de-orientation and model-based polarization decomposition algorithms in backscatter energy analysis, were analyzed.

It has been proven that when observing Masson pine stands vertically, ground scattering dominated backscattering energy in the C- and X-bands, while the Ku-band signal exhibited greater sensitivity to the Masson pine canopy. For side-looking data, the scene backscattering energy was affected by the incident angle, particularly in the cross-polarization mode, where the energy increased with larger incident angles. The interplay among canopy scattering, canopy extinction, and ground scattering resulted in a complex relationship between backscattering energy and canopy density. The orientation correction to polarization scattering matrix can concentrate the energy in the co-polarization mode, thereby reducing the impact of the incident angle on the backscattering energy and the energy decomposition errors caused by the Freeman–Durden model. In addition, even if the model-based polarization decomposition maintained the energy consistency before and after decomposition, the volume scattering energy still showed a complex relationship with the canopy density. In view of the complex interaction between microwave signals and canopy structure, the inversion methods based on physical models should be paid more attention than the empirical models to ensure the accuracy and reliability of forest parameter inversion.

## Figures and Tables

**Table 1:** Measurement indices and accuracies of the platform.

Parameter	Measuring Range and Accuracy
Range of incidence angel (°)	0~90
Accuracy of incidence angel (°)	0.01
Rotation range of turntable (°)	0~360
Accuracy of turntable rotation (°)	0.01
Frequency range (GHz)	0.8~20
Signal-to-noise ratio (dB)	−60

## Data Availability

The data presented in this study are available on request from the corresponding author due to the requirements of the data measurement contract.
